# Seasonal and spatial patterns of microbial diversity along a trophic gradient in the interconnected lakes of the Osterseen Lake District, Bavaria

**DOI:** 10.3389/fmicb.2015.01168

**Published:** 2015-10-28

**Authors:** Katrin Zwirglmaier, Katharina Keiz, Marion Engel, Juergen Geist, Uta Raeder

**Affiliations:** ^1^Aquatic Systems Biology Unit, Limnological Research Station Iffeldorf, Department of Ecology and Ecosystem Management, Technical University of MunichMunich, Germany; ^2^Helmholtz Zentrum München, Scientific Computing Research Unit, German Research Center for Environmental HealthNeuherberg, Germany

**Keywords:** microbial ecology, freshwater lakes, trophic gradient, niche adaptation, 454 amplicon sequencing, bacterial diversity, Osterseen Lake District

## Abstract

The Osterseen Lake District in Bavaria consists of 19 small interconnected lakes that exhibit a pronounced trophic gradient from eutrophic to oligotrophic. It therefore presents a unique model system to address ecological questions regarding niche adaptation and Baas Becking's long standing hypothesis of “everything is everywhere, but the environment selects.” Here, we present the first assessment of the microbial diversity in these lakes. We sampled the lakes in August and December and used 454 pyrosequencing of 16S rRNA amplicons to analyze the microbial diversity. The diversity patterns between lakes and seasons were compared and the bacterial community composition was correlated with key chemical and physical parameters. Distinct patterns of bacterial diversity only emerged at the level of individual OTUs (operational taxonomic units), but not at the level of the major bacterial phyla. This emphasizes the high functional and physiological diversity among bacterial species within a phylum and calls for analysis of biodiversity at the level of OTUs in order to understand fine-scale biogeography. We were able to identify a number of cosmopolitan OTUs as well as specialist OTUs that were restricted to certain lakes or seasons, suggesting adaptation to specific ecological niches.

## Introduction

Bacterial community composition in lake ecosystems is influenced by a number of chemical and physical environmental parameters, as well as by biotic factors. Over the years, numerous studies have analyzed the bacterial diversity and influencing factors in individual lakes (e.g., Zwisler et al., 2003; Shade et al., 2007; Garcia et al., 2013; Gies et al., 2014), or compared lakes of different trophic states (e.g., Van der Gucht et al., 2005; Boucher et al., 2006; Crump et al., 2007; Schiaffino et al., 2011; Liu et al., 2015). A common problem with comparing bacterial diversity in different lakes is that, depending on the geographical distance of the lakes, they will be subject to different climatic influences (temperature, wind, rainfall, UV radiation), different geological settings (age and origin of the lake, structure of the sediment and shore line), hydrological properties (size and shape of the water body, water retention time, vertical mixing) and anthropogenic influence on the lake itself and its catchment area. These differences can mask the effect of individual parameters, such as nutrient concentration and impede the understanding of underlying ecological principles.

We introduce here the Osterseen Lake District, a group of 19 small, interconnected lakes in Bavaria, Germany, that exhibit a natural gradient of nutrient concentrations and present a unique model system for addressing ecological questions. Chemical and physical properties of the lakes, as well as macrophytes and diatom diversity have been monitored for the last 30 years. The lakes are already being used as a model system for macrophytes and diatoms and two ecological indices for the evaluation of water quality, the macrophyte index (Melzer, 1999) and diatom index (Seele et al., 2000) have been developed here. Both indices are incorporated in the European water framework directive (Schaumburg et al., 2011). Here, as a first step for microbial ecology based studies on this lake system, we present the first comprehensive analysis of the microbial diversity in these lakes and link that to the physical and chemical gradients.

## Materials and methods

### Sampling site osterseen lake district

**Figure 1:**
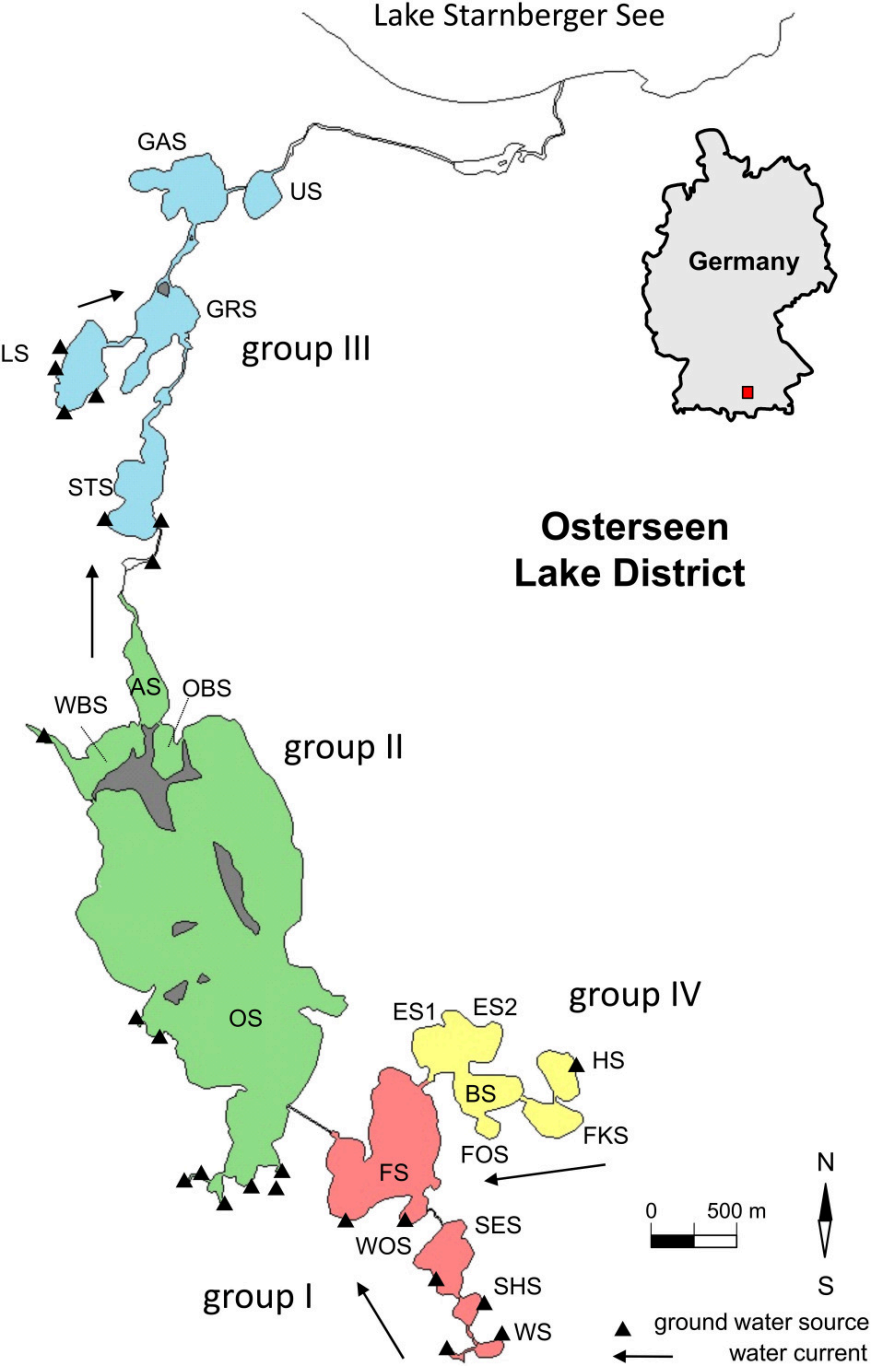
**Map of the Osterseen Lake District**. Abbreviations of the lake names are listed in Table 1.

**Figure 2:**
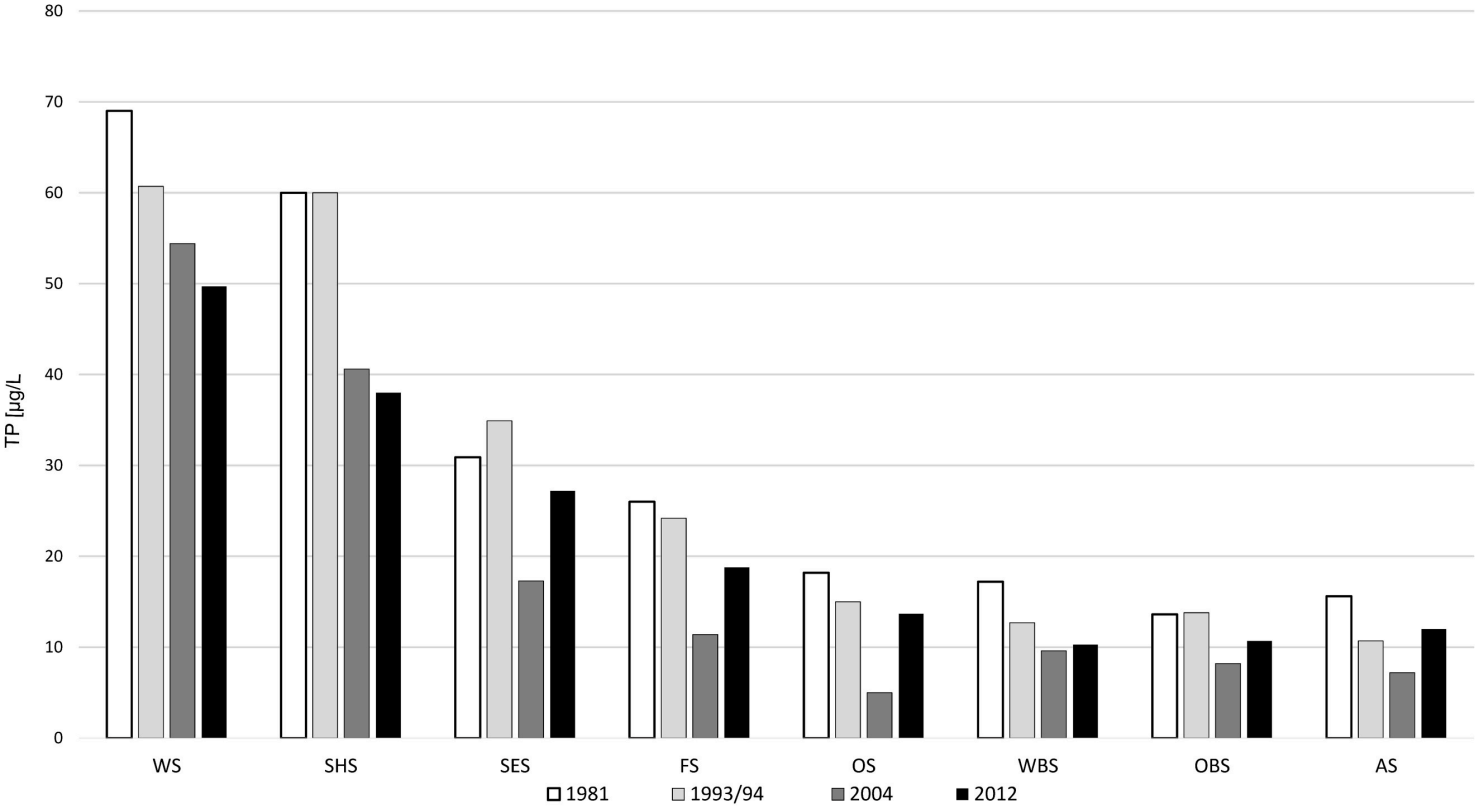
**Total phosphate concentrations in the main chain of lakes over the last four decades**.

The Osterseen Lake District consists of a group of 19 small lakes located in the Bavarian pre-alpine region ca. 50 km south of Munich (Figure 1 and Table 1). The lakes are kettle lakes of glacial origin and are classed as hard-water lakes, characterized by high calcium carbonate concentrations and slightly alkaline pH (ranging from 7.5 to 8.8). All lakes are interconnected by natural channels and to some extent by water bearing layers in the underground. They are fed exclusively by groundwater (Figure 1). An altitude difference of 10 m between the southernmost and northernmost lakes leads to a weak south-north current along the main chain of lakes (Figure 1). The water eventually drains into Lake Starnberg, which lies directly north of the Osterseen Lake District. Anthropogenic influence (municipal wastewater from the village Iffeldorf at the southernmost Lake Waschsee and agricultural use of the surrounding land) has resulted in heavy eutrophication of the southernmost lakes during the twentieth century. Dilution effects along the chain of lakes following the current and differences in the nutrient load of the ground water at several points along the chain have created a unique trophic gradient from eutrophic to oligotrophic conditions. A municipal wastewater treatment plant built in 1983 and changes in the agricultural use of the surrounding land have drastically reduced the nutrient influx and gradually decreased the nutrient load, but the trophic gradient is still observed (Figure 2). The lakes and surrounding area have been declared a nature reserve in 1981.

**Table 1:** **List of lakes in the Osterseen Lake District**.

**Lake**	**Abbr**.	**Lake group**	**Trophic state**	**Surface area (ha)**	**Volume (× 1000 m^3^)**	**Max. depth (m)**
Waschsee	WS	I	Eutrophic	0.85	25.6	5.4
Schiffhüttensee	SHS	I	Eutrophic	1.17	40.8	6.6
Sengsee	SES	I	Eutrophic	5.45	387.9	14.6
Wolfelsee	WOS	I	Mesotrophic	1.06	32.6	5.8
Fohnsee	FS	I	Mesotrophic	21.19	2298.3	23.7
Ostersee	OS	II	Meso-oligotrophic	117.63	14000.0	29.7
Östlicher Breitenauersee	OBS	II	Meso-oligotrophic	2.39	160.0	15.6
Westlicher Breitenauersee	WBS	II	Meso-oligotrophic	6.09	352.0	17.1
Ameisensee	AS	II	Meso-oligotrophic	3.76	346.0	18.9
Stechsee	STS	III	Oligotrophic	7.54	486.0	15.2
Gröbensee	GRS	III	Oligotrophic	6.07	353.0	15.2
Lustsee	LS	III	Oligotrophic	5.92	371.0	18.0
Gartensee	GAS	III	Oligotrophic	7.46	389.0	13.7
Ursee	US	III	Oligotrophic	2.21	111.5	11.8
Eishaussee	ES1^*^/ES2^*^	IV	Mesotrophic	7.69	511.0	19.1
Bräuhaussee	BS	IV	Mesotrophic	5.11	295.0	12.5
Forchensee	FOS	IV	Meso-oligotrophic	0.92	30.0	8.2
Fischkaltersee	FKS	IV	Mesotrophic	3.28	191.8	11.4
Herrensee	HS	IV	Oligotrophic	3.00	148.0	10.7

* *ES1, meromictic western basin; ES2, dimictic eastern basin*.

In previous studies, the lakes have been divided into four groups based on their position, hydrological properties and nutrient load (Figure 1 and Table 1, Zorell, 1941; Melzer, 1999):

– Group I

These are the five southernmost lakes, closest to the village Iffeldorf. They have the highest nutrient load within the Osterseen Lake District, ranging from eutrophic (Lake Waschsee and Schiffhüttensee) to mesotrophic (Lake Fohnsee). They are heavily influenced by groundwater influx. The surrounding land consists mostly of meadows, some of which are used as pastures.

– Group II

This group of four lakes follows the current of the main chain of lakes and includes the largest and deepest lake of the Osterseen Lake District, Lake Grosser Ostersee, which is 2.15 km long and 0.83 km wide with a maximum depth of 29 m. The lakes in this group are less influenced by groundwater sources. Based on their nutrient load they are classed as meso-oligotrophic. They are mostly surrounded by forest. Particularly Lake Grosser Ostersee, and to a lesser extent Lake Ameisensee and the Western and Eastern Lake Breitenauersee are characterized by very high CaCO_3_ concentrations, which results in biogenic CaCO_3_ precipitation in summer (Gruenert and Raeder, 2014), leading to a distinct turquoise water color and low Secchi depths.

– Group III

The five lakes in this group are all oligotrophic. They are surrounded by a wide reed belt and situated in a peat bog. Lakes Stechsee, Gröbensee, Gartensee, and Ursee form part of the main chain and are characterized by a brownish water color due to wash out of humic substances from the peat. Lake Lustsee forms a side arm that drains into the main chain at Lake Gröbensee. Lake Lustsee is the only one of the northern lakes, which is heavily influenced by groundwater sources. In contrast to the other four northern lakes, its water is blue and very clear, with summer Secchi depths often exceeding 10 m.

– Group IV

These five lakes form a side chain, which feeds into the main chain at Lake Fohnsee. The trophic level of these lakes ranges from oligo- to mesotrophic. Water flows from Lake Herrensee, which is the only one in this group with a groundwater source, through lakes Fischkaltersee, Bräuhaussee, Eishaussee and then into Lake Fohnsee. Noteworthy in this group is the western basin of Lake Eishaussee. It is meromictic and therefore permanently anoxic below ca. 8 m.

### Sampling and *in-situ* measurements

Physical parameters (temperature, pH, O_2_ concentration and conductivity) were measured in 1 m steps using a multi parameter probe Multi 350i (WTW, Weilheim, Germany). Water samples were taken in August and December 2012 using a Ruttner sampler. For bacterial DNA extraction a total of 1 L of water from each lake was used, mixing samples taken in 1 m steps from the entire water column over the deepest point of the lake. The water was filtered through 0.2 μm, 47 mm diameter cellulose nitrate filters (Whatman, UK) within a few hours of sampling. Filters were stored at −20°C until DNA extraction. For chemical analysis (total P, NO_3_-N, NH_4_-N), 0.5 L water samples were taken in 2 m steps (1 m steps for the shallow lakes WS, SHS, and WOS) from the entire water column in August during stratification. In December, since the physical measurements indicated a mixed water column, only one mixed water sample from the top 4 m was analyzed.

### Water chemistry

The concentrations of nutrients were measured spectrophotometrically using standard methods. Total phosphorus and NH_4_-nitrogen were measured according to DEV (2013), NO_3_-nitrogen was measured according to Navone (1964).

### DNA extraction

DNA was extracted from the filters using a phenol/chloroform-based protocol. Filters were placed in 15 ml tubes and cells on the filters were lysed in 2 ml DNA lysis buffer (0.25 M Tris, 25% sterile filtered sucrose) and 20 μl lysozyme (50 mg/ml) at 37°C. After 30 min, 200 μl SDS (10% w/v) and 15 μl Proteinase K (20 mg/ml) were added and samples were incubated for another 2 h at 37°C, followed by 30 min at 50°C. After this, 2 ml of phenol/chloroform/isoamylalkohol 25:24:1 were added, the samples were shaken vigorously and then centrifuged 5 min at 3300 × g. The upper aqueous phase was carefully transferred to a new 15 ml tube, 2 ml of chloroform were added, the tube was shaken and centrifuged again for 5 min at 3300 × g. The upper aqueous phase was transferred to a fresh tube and DNA was precipitated with 2 volumes of ethanol and 0.1 volumes of 3 M sodium acetate, followed by 45 min centrifugation at 3300 × g and 4°C. The DNA pellet was washed with 70% ethanol and resuspended in TE buffer (10 mM Tris, 1 mM EDTA, pH 8.0). DNA was stored at -20°C until further use.

### 454 Pyrosequencing

Part of the 16S rRNA gene was amplified using universal primers S-D-Bact-0785-a-S-18 and S-^*^-Univ-1392-a-A-15 1392R (Klindworth et al., 2013), which cover variable regions V5–V8. Sequencing was done unidirectionally from the forward primer. PCR conditions were: initial denaturation 94°C, 3 min, followed by 20 cycles of 94°C, 45 s, 55°C, 30 s, 72°C, 60 s and a final elongation step of 72°C, 5 min, using Accuzyme Taq (Bioline, Cambridge, UK). All PCR reactions were done in triplicate and amplicons were pooled before the purification step. PCR products were purified using the Invitek MSB PCRapace column purification kit (Stratec, Birkenfeld, Germany) according to the manufacturer's protocol. Following the 454 sequencing guidelines for unidirectional sequencing, primer sequences were extended by the adapter sequences A and B for forward and reverse primers respectively, the key sequence, and in the case of the forward primer by the multiplex indices (MID) in a second PCR step with the same PCR conditions as above, again done in triplicates. Samples were run on a 0.8% agarose gel, bands were cut out and DNA was extracted from the gel using Qiagen GelExtraction kit (QIAGEN, Hilden, Germany) according to the manufacturer's protocol. Samples were quantified, pooled and then sequenced with 454 FLX Titanium at LGC (Berlin, Germany) and Helmholtz Zentrum München (Neuherberg, Germany). Initial data processing was performed using gsRunProcessor v2.9. Sequence data have been submitted to NCBI (BioProject ID PRJNA282452).

### Sequence analysis

Sequence data were processed using a combination of Usearch version 7 (Edgar, 2010) and associated python scripts, mothur version 1.31 (Schloss et al., 2009), SINA (Pruesse et al., 2012), and arb (Ludwig et al., 2004).

Sequence reads were trimmed within mothur to remove barcodes and primers and then converted from fasta to fastq format, also within mothur. Usearch was then used to trim all reads to a length of 500 nt and filter out low quality reads (parameter truncqual = 10). Reads were renamed to include the sample name in the header and pooled with unix commands sed and cat, followed by dereplication of reads, removal of singletons, clustering (at the level of 97% identity) and removal of chimeras, all within Usearch. The resulting representative OTUs were renamed with a python script available from the Usearch website, reads were mapped to the representative OTUs and an OTU table was created using Usearch and associated python scripts. The OTUs were classified using the SINA aligner and classification tool on the Silva website (www.arb-silva.de). Further classification for selected OTUs was done within arb using a custom database for cyanobacterial and chloroplast sequences. Relative abundance of bacterial OTUs was corrected for copy number of the 16S rRNA gene using PICRUSt (Langille et al., 2013).

### Statistical analyses

Statistical analysis, i.e., Shannon diversity, Bray-Curtis distance matrix, ANOVA, Mann-Whitney *U*-test, Pearson correlations, neighbor joining cluster analysis, non-metric multidimensional scaling (NMDS) and canonical correspondence analysis (CCA) was carried out with non-transformed data using PAST v3.06 (Hammer et al., 2001) and plotted with Microsoft Excel (Shannon diversity, NMDS and CCA) and FigTree v1.4.2 (Neighbor joining tree; http://tree.bio.ed.ac.uk/software/figtree). QIIME v1.8 (Caporaso et al., 2010) was used to analyze shared OTUs. Depth profiles of environmental parameters were plotted with Ocean Data View v4.6 (Schlitzer, 2014).

## Results

### Physical and chemical parameters

The established division of the Osterseen Lake District into four distinct lake groups was confirmed by measurements of key physical and chemical parameters in August and December 2012. At both sampling time points, the four lake groups differed significantly (One-way ANOVA) in their concentrations of total phosphorus (TP; *p* = 0.04), NO_3_-nitrogen (*p* < 0.01) and NH_4_-nitrogen (*p* < 0.01), as well as in conductivity (*p* < 0.01). In December, the groups additionally showed significant differences in their temperature (*p* = 0.03) and pH (*p* < 0.01).

**Figure 3:**
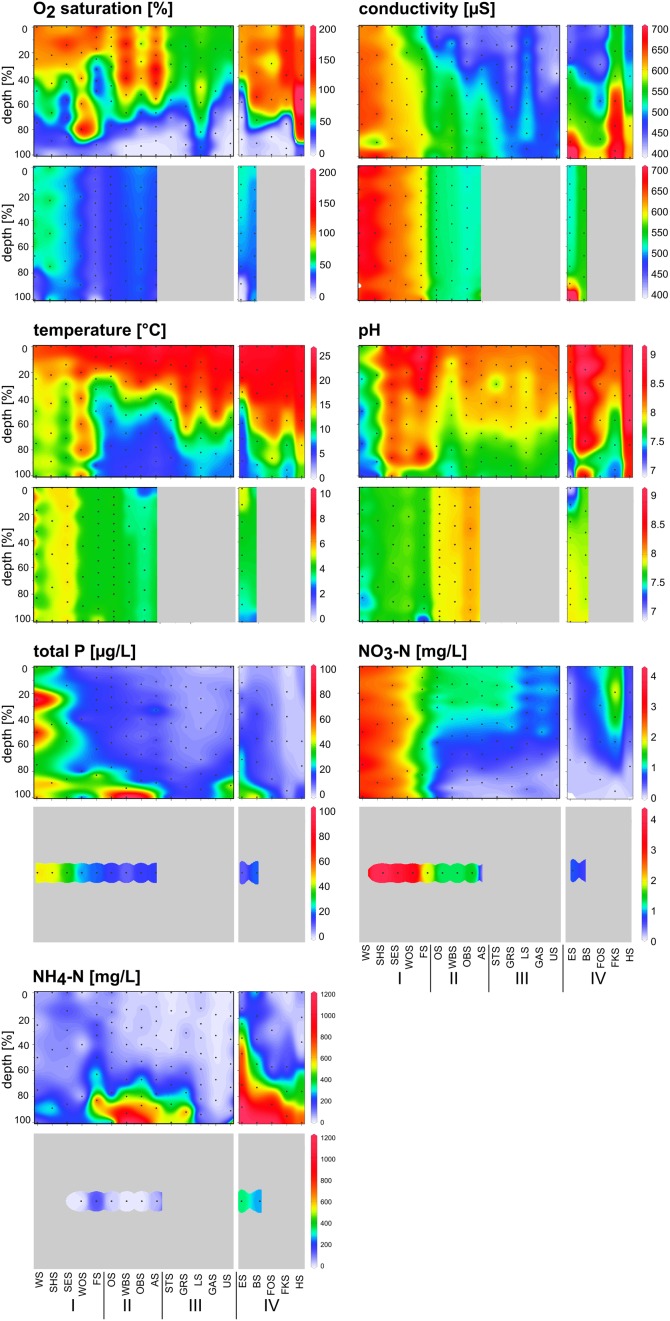
**Depth profiles of chemical and physical parameters along the chain of lakes in summer (upper panel) and winter (lower panel) following the water current south-north (groups I, II, and III) and side chain (group IV)**. Black dots indicate the sampling points. Chemical parameters in winter were measured from one mixed water sample in each lake.

Depth profiles of the measured environmental variables indicated a stratified water column in the summer samples (Figure 3, upper panels). The heavy nutrient load of the group I lakes, particularly the first two lakes, Lake Waschsee and Lake Schiffhüttensee, was evident from the concentrations of TP and NO_3_-N. NH_4_-N was restricted to deeper layers in the water column in the group II lakes and was particularly high in the group IV lakes. The physical parameters temperature, conductivity, pH and O_2_ saturation illustrate the extensive ground water influence into Lake Waschsee and Lake Schiffhüttensee, and to a lesser degree into Lake Lustsee. These lakes had a higher conductivity, lower temperature and pH. The vertical gradients of these parameters were less pronounced. Therefore, the stratification is here less stable compared to the other lakes.

In winter, the physical parameters indicated a completely mixed water column in all sampled lakes, with the exception of the meromictic Lake Eishaussee (Figure 3, lower panels). Due to ice cover, not all lakes could be sampled in winter. The influence of groundwater was again evident in Lake Waschsee and Lake Schiffhüttensee. The relatively constant temperature (8–10°C) of the inflowing groundwater throughout the year and its large quantity relative to the lake volume affected the thermal conditions of these lakes. Lake Waschsee and Lake Schiffhüttensee were markedly colder than the other lakes during summer, but their temperature decreased only slightly in winter so at that point they were warmer than the other lakes. Conductivity and pH showed a sharp split between the group I and group II lakes, which is less evident in summer.

### Microbial diversity

Microbial diversity was assessed by 454-amplicon sequencing of a fragment of the 16S rRNA gene. A total of 31 samples were sequenced. This includes 20 samples collected in summer (August) 2012, i.e., all 19 lakes with two samples from Lake Eishaussee (sample ES1 from the meromictic western basin and sample ES2 from the dimictic eastern basin) and 11 samples collected in winter (December) 2012. The northern lakes (group III) and several of the group IV lakes could not be sampled in December due to ice cover. After a quality control, 128,671 sequence reads were analyzed.

**Figure 4:**
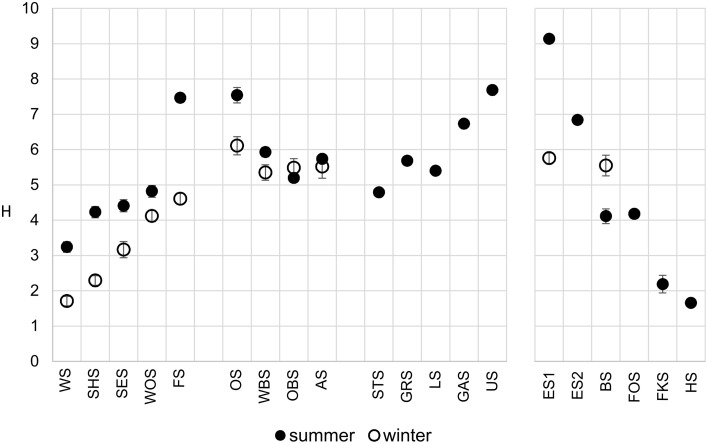
**Comparison of OTU-based microbial Shannon diversity in summer (filled circles) and winter (open circles)**. Error bars denote 95% confidence interval.

The Shannon diversity index of the microbial communities increased along the main chain of lakes (Figure 4), following the trophic gradient from eutrophic to oligotrophic. Diversity in the winter samples was markedly lower, particularly in the group I lakes, but followed the same trend. The middle lakes (group II), which were very similar in their physical and chemical properties (Figure 3), showed similar Shannon diversity values. The group IV lakes, following the water current from the oligotrophic Lake Herrensee to the mesotrophic Lake Eishaussee showed a steep increase in Shannon diversity.

The total number of OTUs found in the dataset was 663. This includes 41 OTUs that were identified as chloroplasts, indicative of eukaryotic phytoplankton. These were deliberately included for some aspects of the analysis to convey an idea of the distribution of eukaryotic (chloroplasts) and prokaryotic (cyanobacteria) phytoplankton. It should be noted, however, that since eukaryotic algae can possess large numbers of chloroplasts and multiple genome copies within each chloroplast, varying with species and cell cycle stage, the number of chloroplast sequences cannot be directly compared with the number of prokaryotic sequences to evaluate relative abundances. Nevertheless, trends of the number of sequence reads of individual chloroplast OTUs, as well as the ratio of chloroplast to cyanobacterial reads are still informative as an indication of who is contributing to primary production.

### Trends of major bacterial phyla

**Figure 5:**
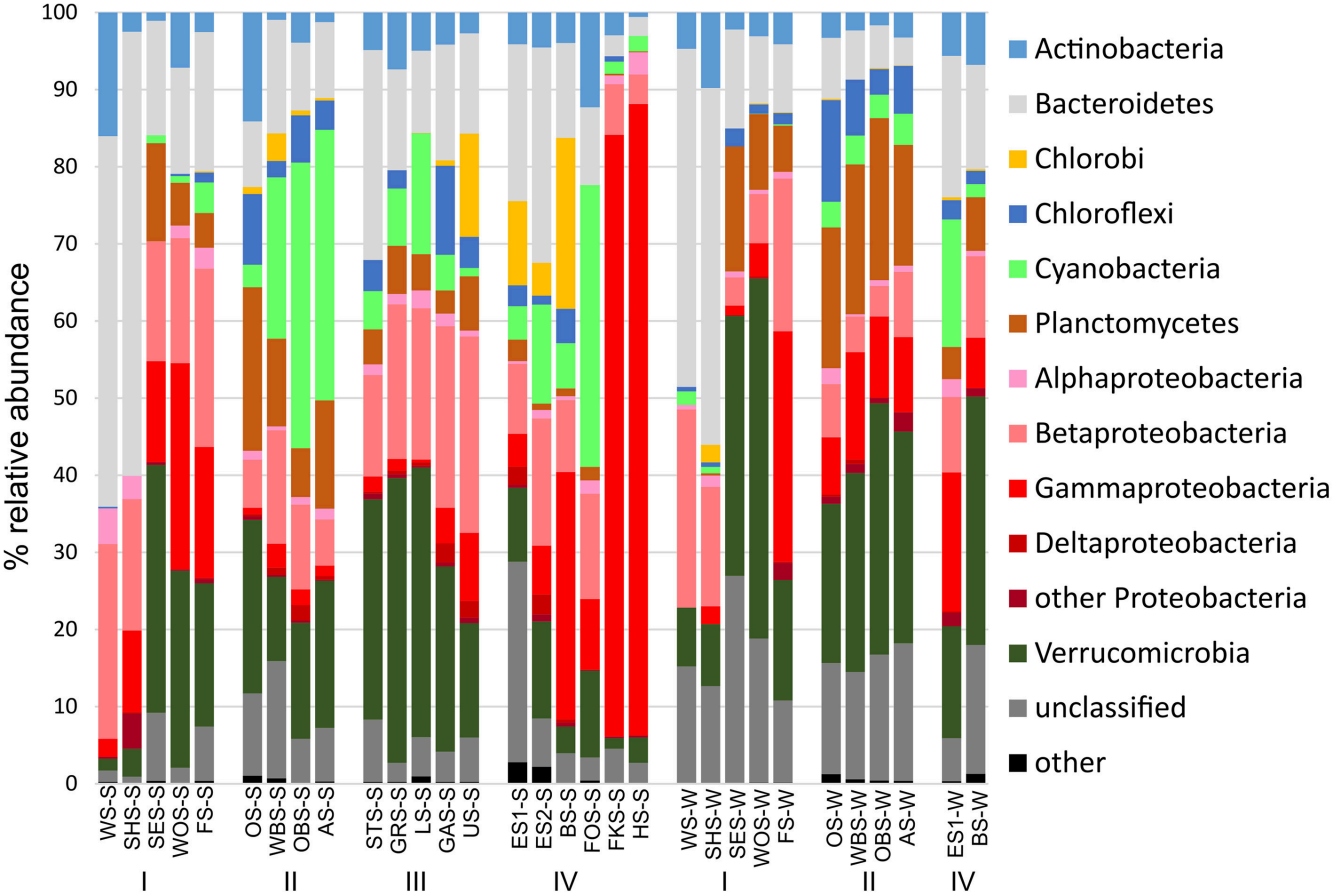
**Relative abundance of the major bacterial phyla**. Chloroplast reads were excluded from the dataset.

**Figure 6:**
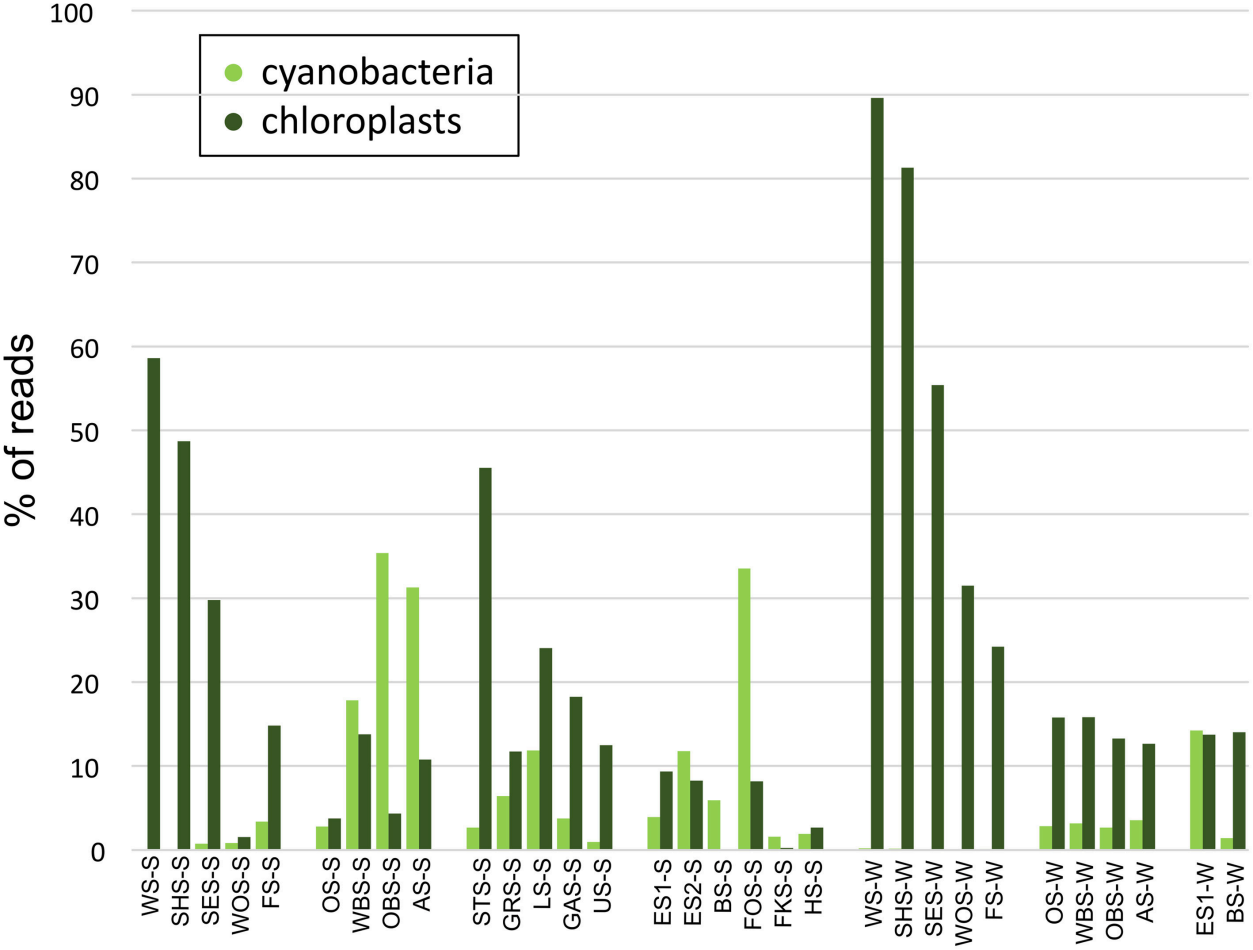
**Relative abundance of cyanobacteria vs. chloroplast sequence reads**.

At the phylum level, only few patterns of differential distribution across the lakes were apparent (Figure 5). Notable are the high abundances of Bacteroidetes (44–57% of bacterial sequences) in Lake Waschsee and Lake Schiffhüttensee in both summer and winter and the striking dominance of Gammaproteobacteria in Lake Herrensee (82%) and Lake Fischkaltersee (78%). Verrucomicrobia were ubiquitous in most lakes except the eutrophic Lake Waschsee and Lake Schiffhüttensee and the mesotrophic group IV lakes. Cyanobacteria were virtually absent in the eutrophic-mesotrophic group I lakes and generally had a lower abundance in winter. They had their highest abundances in summer in the group II lakes (up to 37%) and Lake Forchensee (group IV). Chloroplasts, i.e., eukaryotic phytoplankton, showed a contrasting distribution to cyanobacteria (Figure 6), with the highest abundance of sequence reads in the eutrophic lakes and a gradual decrease toward the oligotrophic lakes, as well as a generally higher abundance in winter compared to summer. The two groups were negatively correlated (Pearson = −0.349).

### Distribution patterns of individual operational taxonomic units (OTUs)

**Figure 7:**
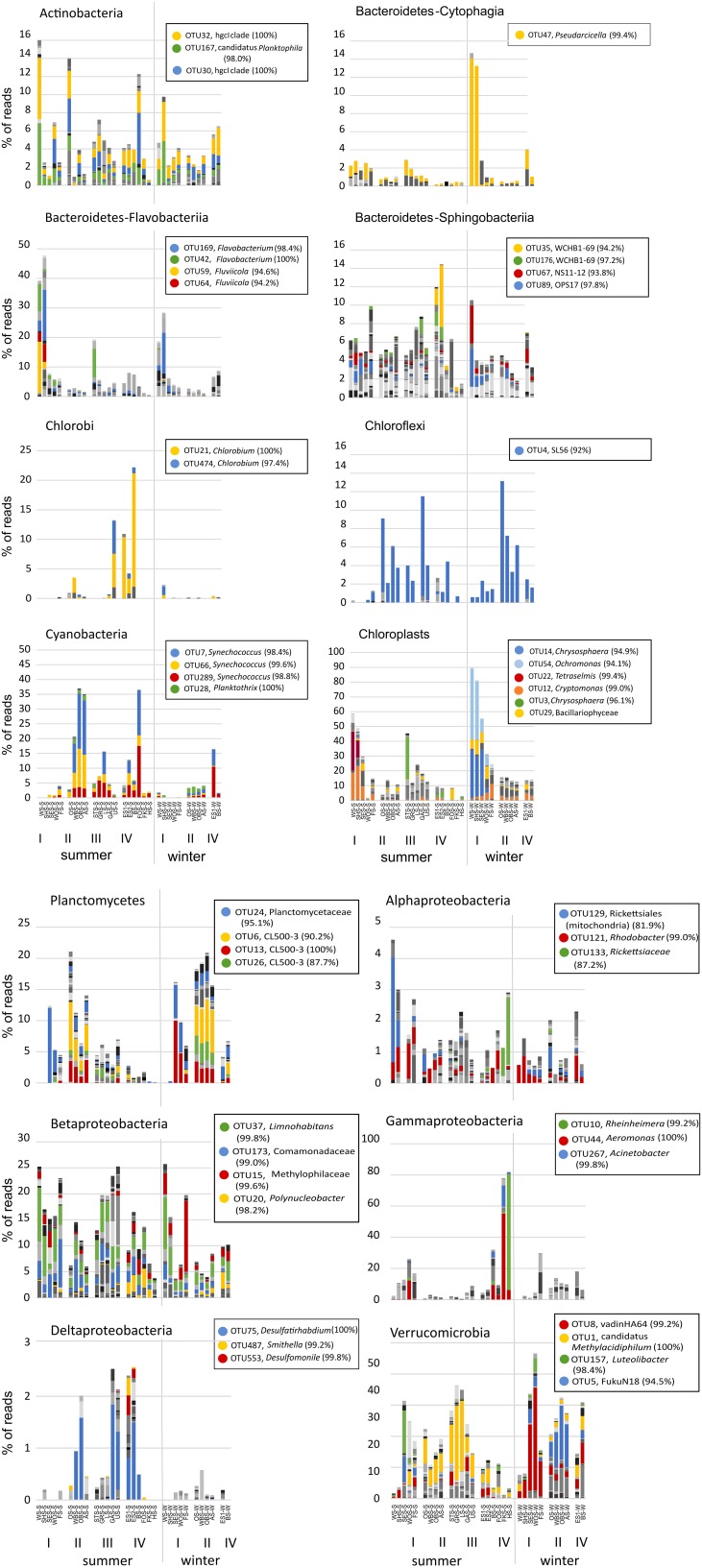
**Distribution patterns of OTUs within individual bacterial phyla across all lakes in summer and winter**. Colors represent different OTUs at the level of 97% sequence identity. For selected dominant OTUs the classification based on the Silva 119 reference database and % sequence identity with the closest known relative are shown in the legend. A full OTU table with the relative abundance and classification of all OTUs is given in Supplementary Table ST1. Chloroplast reads were excluded from the dataset in all panels except the chloroplast panel. Note the different scales in the panels.

A more distinct picture of diversity patterns emerged at the level of individual OTUs. Figure 7 shows the distribution of OTUs within individual bacterial phyla across all lakes and seasons. A full OTU table with relative abundance values and taxonomic classification of the OTUs is given in Supplementary Table ST1. OTUs were defined at the level of 97% sequence identity and abundances were corrected for rRNA gene copy number with PICRUSt.

While the total abundance of Bacteroidetes in the eutrophic lakes Waschsee and Schiffhüttensee was similar in both summer and winter, there were strong seasonal differences within the Bacteroidetes classes Flavobacteriia, Sphingobacteriia and Cytophagia in the abundances of individual OTUs. In summer a range of Flavobacteriia OTUs were the main contributors to the Bacteroidetes community in these two lakes, while in winter their abundance decreased sharply. Instead the abundance of one Cytophagia OTU (OTU 47, which has 99.4% sequence identity with *Pseudarcicella*) increased markedly. Across all lakes Sphingobacteriia were highly diverse, in contrast to Flavobacteriia and Cytophagia, which were represented by only few OTUs. Chlorobi and Chloroflexi populations were each dominated by just one OTU, identified as *Chlorobium* (100% sequence identity) and putatively SL56 (92.0% sequence identity), respectively. Chlorobi appeared only in summer, mainly in the group IV lakes and Lake Ursee, while Chloroflexi were found in both summer and winter. Cyanobacteria showed only little diversity. The community was dominated by three OTUs, all classified as *Synechococcus*, which disappeared in winter in most lakes except the western basin of Lake Eishaussee. The group II lakes also had low concentrations of a Cyanobacteria OTU classified as *Planktothrix*, which was found in both summer and winter. Chloroplasts, i.e., eukaryotic phytoplankton, showed strong seasonal differences in their community composition and a markedly higher abundance in the eutrophic lakes, which were almost devoid of cyanobacteria. The dominant chloroplast OTUs in summer were classified as Cryptophyceae (OTU12), with *Cryptomonas curvata* as the closest cultured relative (99.0% identity) and Prasinophyceae (OTU 22) with *Tetraselmis cordiformis* as the closest cultured relative (99.4% identity). In contrast to that the dominant chloroplast OTUs in winter were Chrysophyceae (OTU 54 with 94.1% identity with *Ochromonas distigma* and OTU14 with 94.9% identity with *Chrysosphaera* sp. CCMP296) and Bacillariophyceae. Planctomycetes showed less seasonal variation, but stronger associations with specific lake groups, particularly the group II lakes, which had high abundances of several OTUs (OTUs 6, 13, 26) classified as Phycisphaeraceae. Within the phylum Proteobacteria, representatives of the class Alphaproteobacteria appeared in low abundance (<5%) in all lakes and seasons. Notable is OTU 133 (Rickettsiaceae-Orientia), which showed a strong preference for lakes Fischkaltersee and Herrensee. The community of Betaproteobacteria was highly diverse and contributed up to 25% to the total bacterial sequences. The dominant OTUs 37, 173, and 15 were classified as *Limnohabitans* (99.8% sequence identity), Comamonadaceae (99.0%), and Methylophilaceae (99.6%). OTU 20 (98.2% identity with Polynucleobacter) was mostly restricted to the group IV lakes. Deltaproteobacteriaceae only appeared in low abundances <3% with the dominant OTU 75 (*Desulfatirhabdium*) only found in the summer samples. The extreme dominance of Gammaproteobacteria in Lake Fischkaltersee and Lake Herrensee is based on mainly one OTU (OTU 10, classified as *Rheinheimera*) in Lake Herrensee, and two OTUs (OTU44, classified as *Aeromonas* and OTU267, classified as *Acinetobacter*) in Lake Fischkaltersee. Verrucomicrobia OTUs exhibited strong seasonal differences and in winter also some preferences for specific lake groups. While OTU1 (classified as *Methylacidiphilum*) dominated the Verrucomicrobia community in most lakes in summer, in winter OTU 8 (Opitutae–vadinHA64) and OTU 5 (Chtoniobacterales–FukuN18) dominated the group I and group II lakes, respectively.

### Correlation with environmental parameters

**Figure 8:**
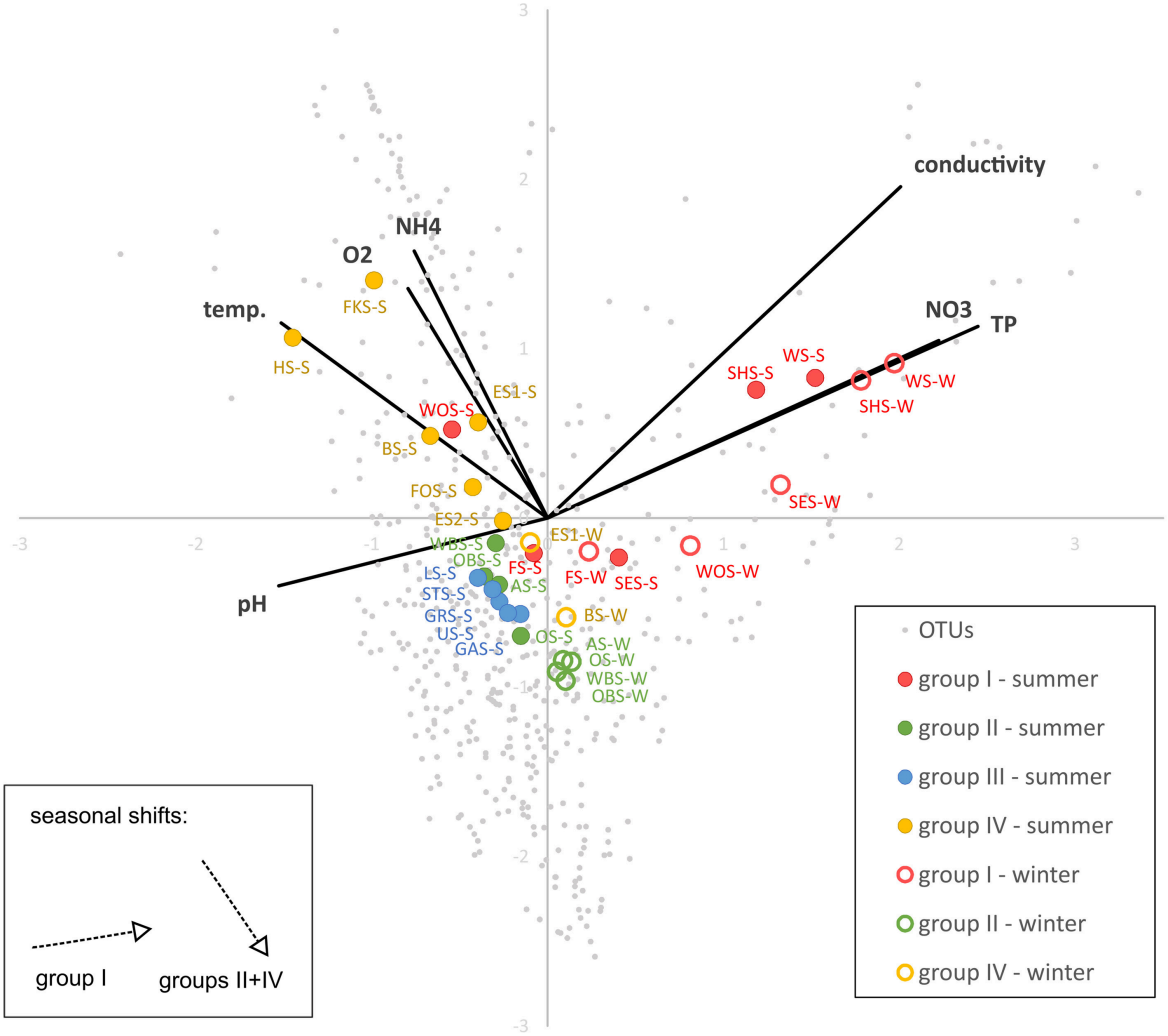
**Canonical correspondence analysis (CCA) showing the correlation of OTU-based microbial community patterns with environmental parameters**. Variance explained by x-axis: 33.4%, y-axis: 22.4%).

CCA (Figure 8) shows a strong positive correlation of the group I lakes with nitrate, phosphate and conductivity and a negative correlation with pH. The lakes align along a gradient of phosphate, nitrate and conductivity following the order of the lakes in the chain. The lakes in group IV, and to a lesser degree groups II and III align along a gradient of ammonia, temperature and oxygen concentrations. They are not substantially separated by their preferences for phosphate, nitrate, conductivity or pH. The winter samples of groups II and IV (no samples from group III available) are all shifted in the same direction, toward lower temperature, oxygen and ammonia concentrations. Conversely, the seasonal shift of the group I lakes is toward higher phosphate, nitrate and conductivity and lower pH, but less influenced by temperature, oxygen and ammonia. The exception in this group is Lake Wolfelsee (WOS), which follows the shift of the group II and IV lakes.

**Figure 9:**
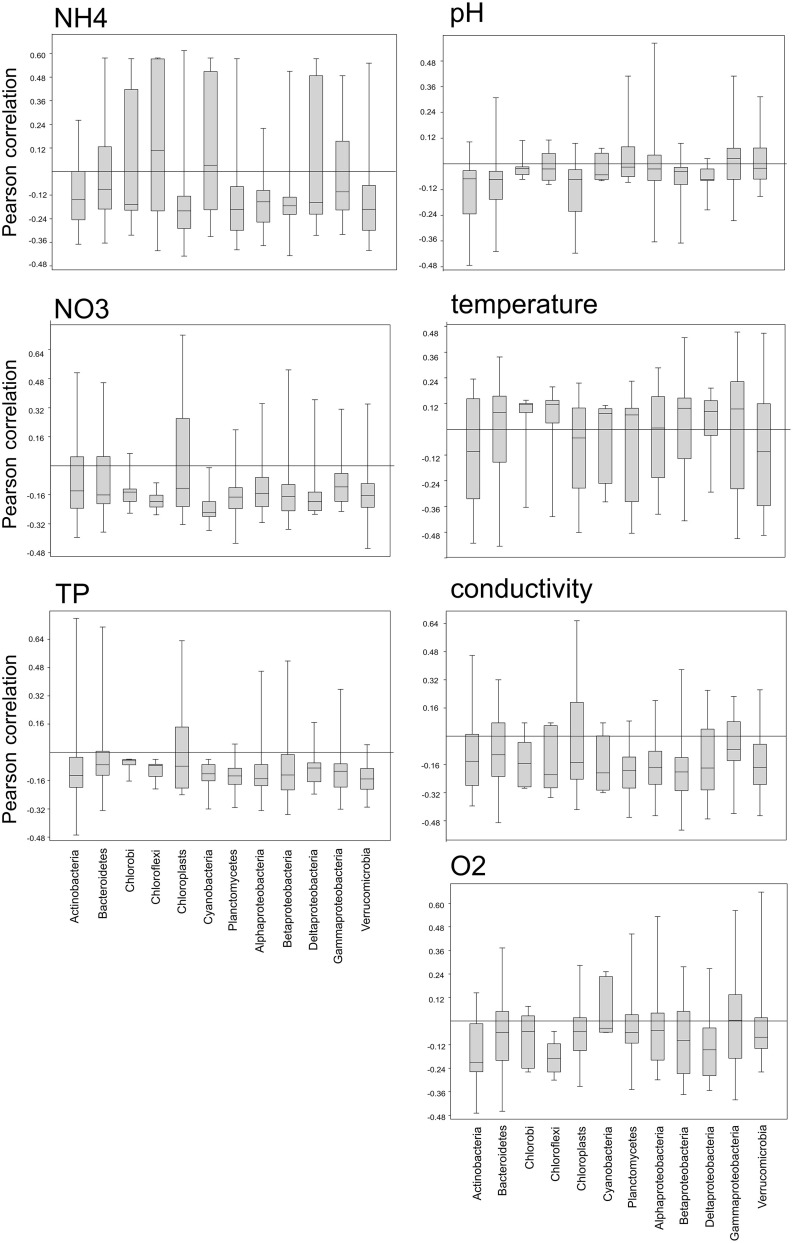
**Box-whisker plots showing the range of positive or negative Pearson correlations of OTUs within a phylum with several environmental parameters**.

We also examined the correlation of OTUs within a phylum with several environmental parameters (Figure 9) in order to gain insights into the range of environmental preferences of organisms classed within the same phylum. All bacterial phyla, but not the chloroplasts, showed a narrow range of slightly negative correlations with phosphorus and nitrate, with a few outliers exhibiting either strong positive or negative correlations. In contrast to that there are distinct differences between the bacterial phyla in their correlation with ammonia. Planctomycetes, Verrucomicrobia, Alpha-, and Beta-proteobacteria, as well as Chloroplasts show a narrow range of negative correlations with ammonia, while OTUs within the Chlorobi, Chloroflexi, Cyanobacteria, and Deltaproteobacteria show a wide range of either positive or negative correlations.

### Beta diversity

**Figure 10:**
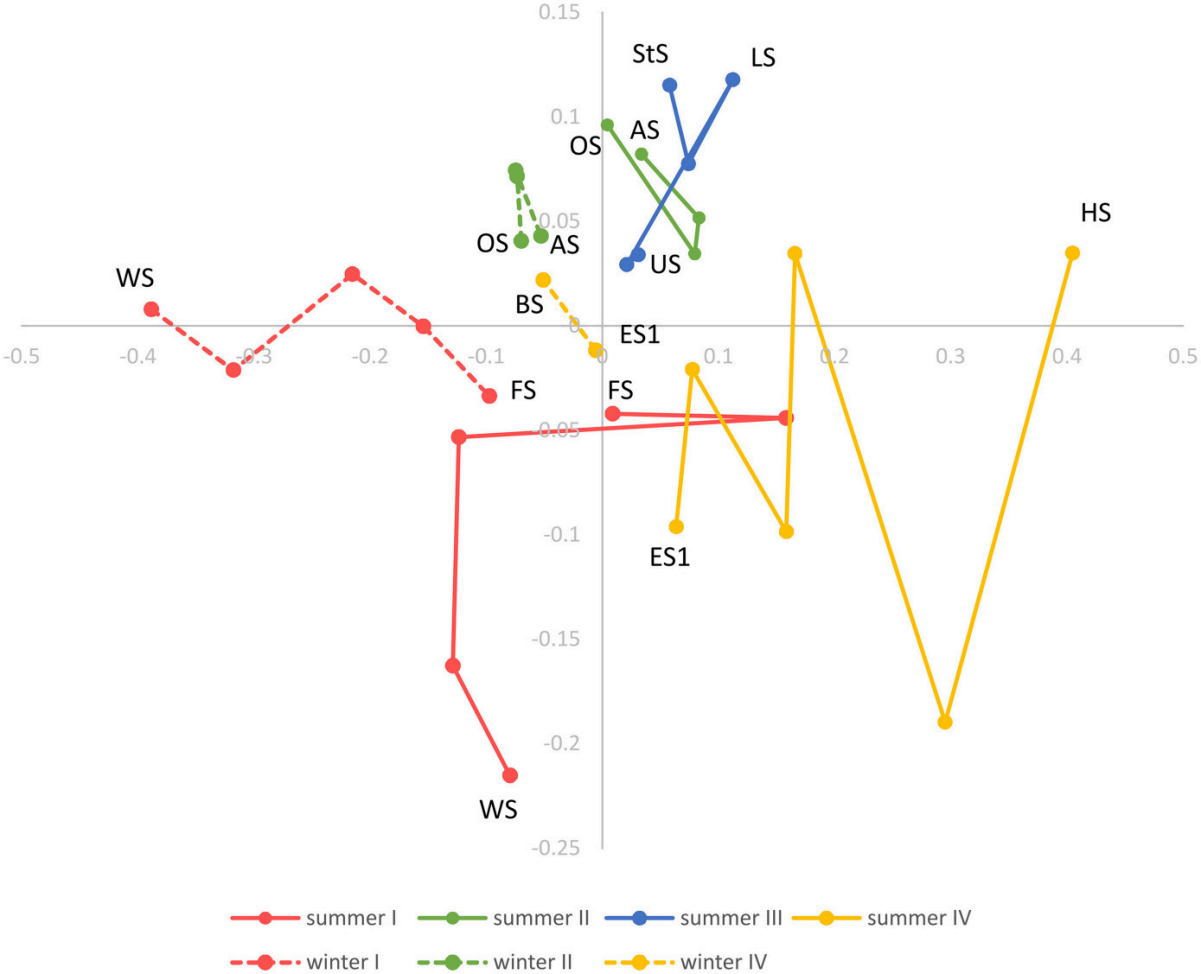
**Non-metric multidimensional scaling (NMDS) based on a Bray-Curtis dissimilarity matrix**. Lakes in each lake group are connected according to their geographical position and following the water current.

Beta diversity across the Osterseen lake system was explored with NMDS and neighbor joining cluster analysis, both based on a Bray-Curtis distance matrix. The NMDS plot (Figure 10) shows increasing dissimilarity of the bacterial population with increasing physical distance of the lakes. The bacterial community composition in the summer samples within and between lake groups II and III is very similar and these lakes cluster close together. In contrast, the lakes within groups I and IV are more spread out in the NMDS plot, indicating greater differences in their bacterial community compositions. This reflects the steep gradients in environmental parameters in lake groups I and IV and the rather moderate environmental differences within and between groups II and III (Figure 3). The positions of the summer samples on the NMDS plot roughly reflect the geographical positions of the lakes on the map (Figure 1), with the southernmost Lake Waschsee and the northernmost Lake Ursee at opposite ends and the side chain of the group IV lakes approaching the northern end of the group I lakes near lake Fohnsee. The positions of the winter samples also reflect the geographical positions of the lakes, but are shifted from the summer samples, without any overlap between summer and winter. The greatest shift between summer and winter samples, i.e., the greatest differences in bacterial community composition, was found for the eutrophic lakes Waschsee and Schiffhüttensee.

**Figure 11:**
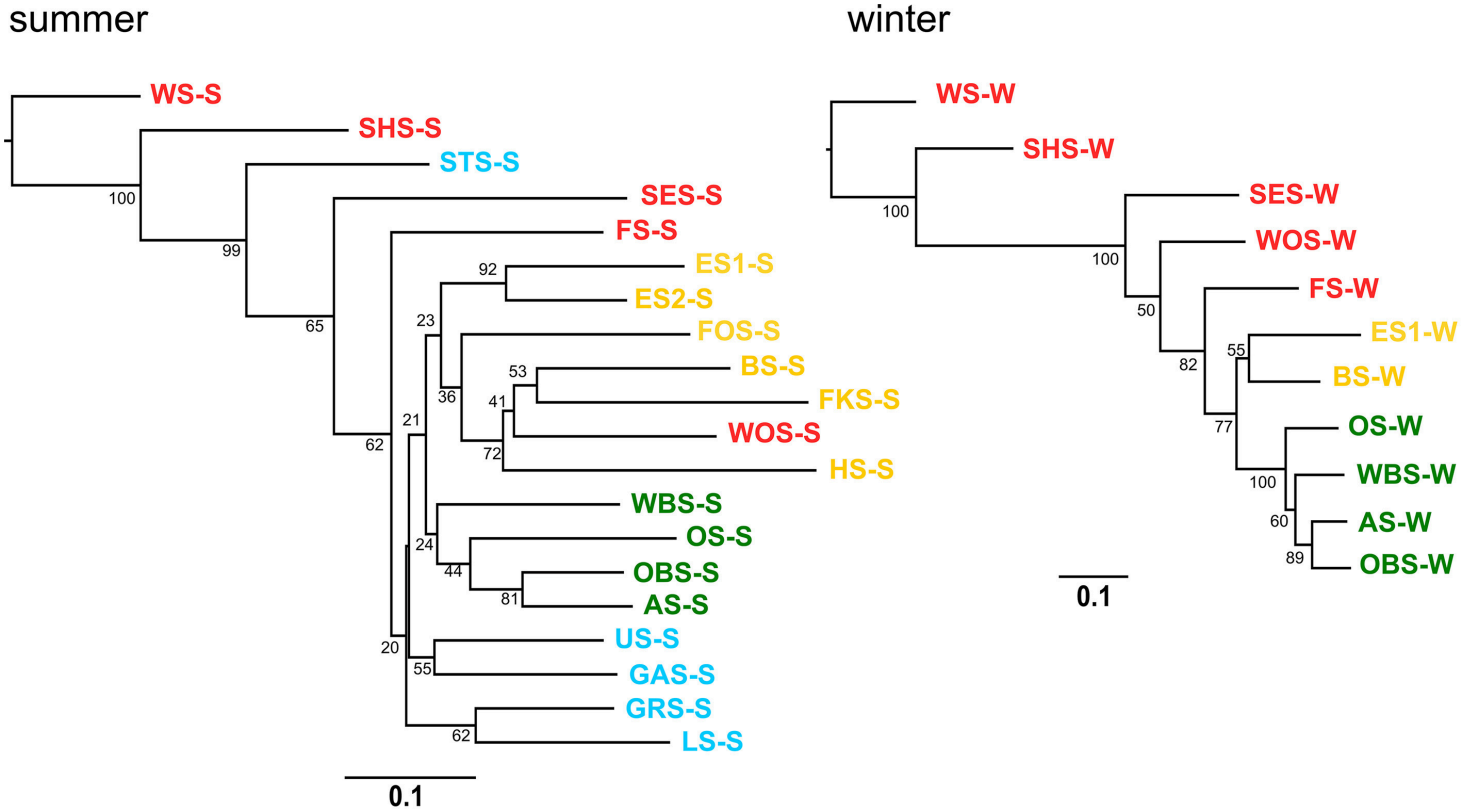
**Cluster analysis–Neighbor joining tree based on Bray-Curtis distance matrix**. Numbers on branches indicate bootstrap values (1000 replicates). Scale bar indicates a distance of 0.1.

A neighbor joining tree (Figure 11) of the winter samples shows clustering of the lakes according to the lake groups and following their geographic location. Lake Waschsee and Lake Schiffhüttensee form an outgroup, corresponding to the distinctly different microbial communities in those two eutrophic lakes (Figure 7). The clustering of the summer samples is less clearcut, but still essentially corresponds to the lake groups and geographical position of the lakes.

### Similarity of the bacterial communities between lakes and seasons

**Figure 12:**
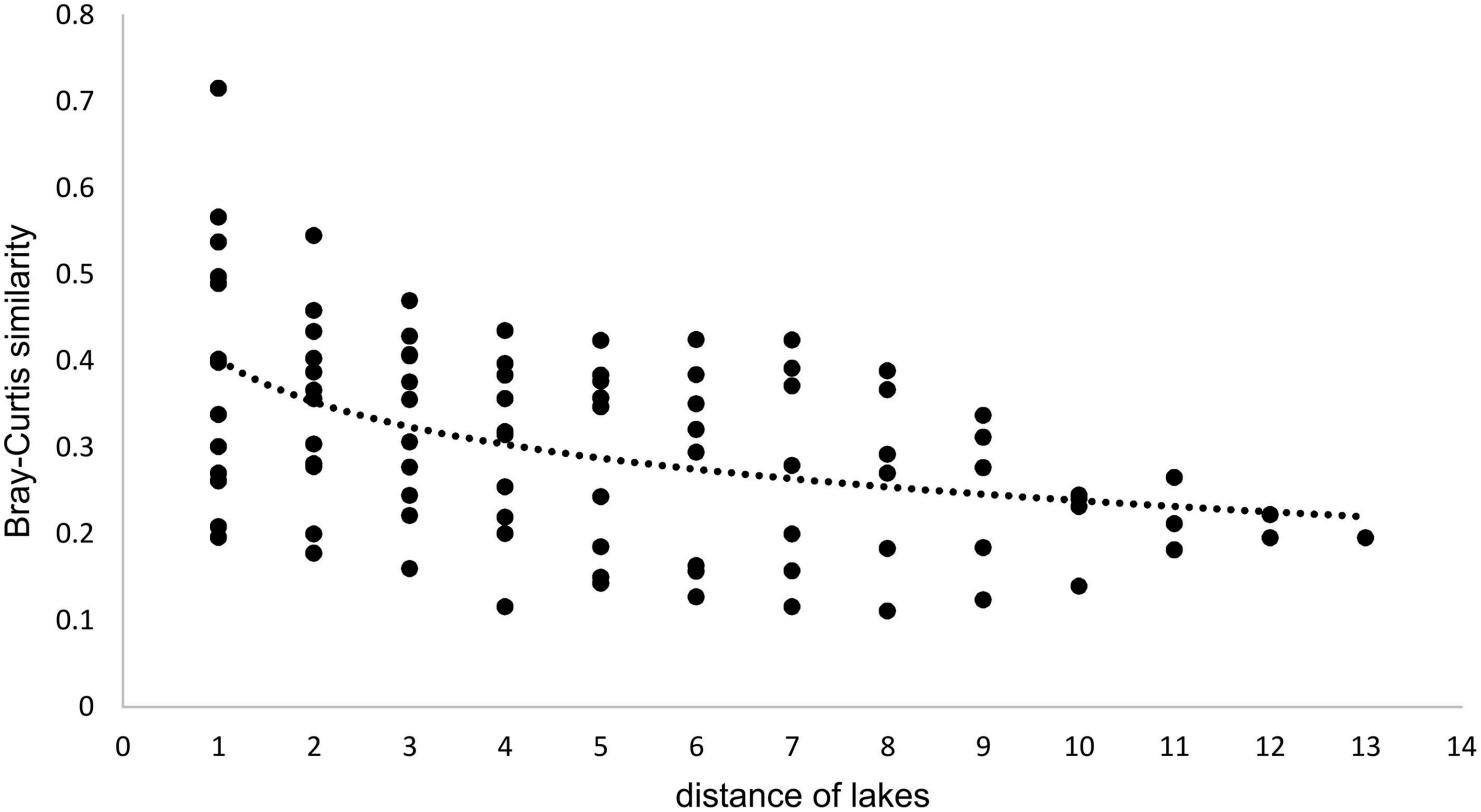
**Distance-decay analysis along the main chain of lakes**. Distances refer to the position of the lakes, i.e., directly adjacent lakes have a distance of 1, lakes that are separated by one lake have a distance of 2, etc…  Regression curve details: *y* = −0.071ln(x) + 0.4018, *R*^2^ = 0.2092.

**Figure 13:**
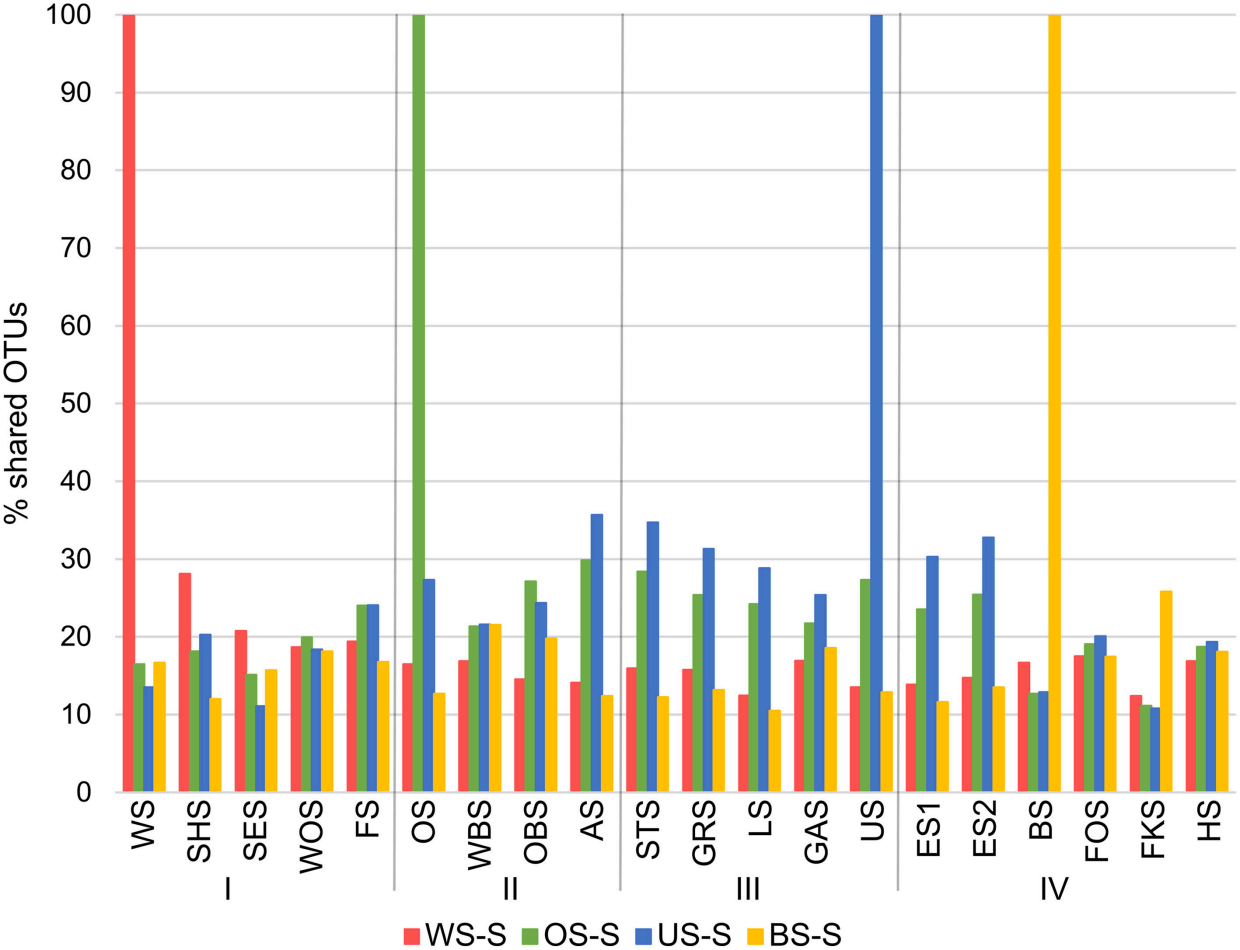
**Percentage of OTUs that representative lakes from each group (summer samples only) share with each lake in the Osterseen Lake District**.

Since all lakes in the Osterseen Lake District are connected and a weak south-north current transports water from the beginning of the chain of lakes (Lake Waschsee) toward the end of the chain (Lake Ursee), a certain amount of overlap between the bacterial communities is to be expected between adjacent lakes and following the current with decreasing similarities the further two lakes are apart. A distance-decay analysis (Figure 12), based on the Bray-Curtis similarity of the microbial communities between pairs of lakes with increasing distance did indeed show a slight decrease in similarity along the main chain of lakes. However, a second approach to look at similarities between the lakes by comparing the percentage of shared OTUs between pairs of lakes revealed that each lake has a very distinct bacterial community. On average, only 21.1% of OTUs (range 9.8–37.6%) are shared between any two lakes in summer and slightly more in winter (24.0% average, range 11.1–38.5%). The composition of the bacterial community also differed considerably between seasons within each lake, with an average of 32.4% shared OTUs between the summer and winter sample of the same lake (range 20.9–49.2%). Nevertheless, neighboring lakes shared slightly, but significantly more OTUs than non-neighboring lakes (Mann-Whitney *U*-test, *p* = 0.01 in summer, *p* = 0.003 in winter). This is visualized in Figure 13, which shows the percentage of OTUs representative lakes from each lake group share with each lake in the lake system.

Four OTUs were present in all lakes in both summer and winter. These were OTU32 (100% identity with Actinobacteria—Sporichthyaceae, hgc cladeI), OTU23 (100% identity with Bacteroidetes—*Flavobacterium*), OTU173 (99.0% identitiy with Betaproteobacteria—Comamonadaceae), OTU37 (99.8% identity with Betaproteobacteria—Comamonadaceae—*Limnohabitans*). The abundance of these OTUs ranged between 0.2 and 10.4% (average 2.1%) of the total bacterial sequence reads.

## Discussion

### Trophic gradient within the lake system—the osterseen lake district as an ecological model system

This study presents the first assessment of microbial diversity in a unique lake system. The trophic gradient along the chain of interconnected lakes make the Osterseen Lake District a valuable study site for addressing ecological questions relating to niche adaptation. Long-term monitoring data of the chemical and physical parameters, as well as diatom and macrophyte distribution from the last four decades have shown the gradient to be stable, but have also provided evidence for the impact and effectiveness of environmental protection measures.

In terms of niche adaptation at the microbial level, the distribution patterns of individual OTUs we found pointed out highly specialized OTUs that were restricted to a certain lake, lake group or season. On the other hand, several cosmopolitan OTUs were identified. Most cosmopolitan OTUs only appeared in low abundances, while OTUs appearing in high abundances were generally restricted to lakes with similar environmental conditions, suggesting specific adaptation to these parameters. Due to the connection between the lakes and the water current, the cosmopolitan OTUs may have been carried over into all lakes, corresponding to the “everything is everywhere” part of Baas Becking's famous hypothesis (Baas Becking, 1934; de Wit and Bouvier, 2006). A caveat, based on the methodology used in our study is that it is not known, whether these cosmopolitan OTUs are actually metabolically active or even alive. However, the restricted localized appearance of other OTUs (which could have just as well been transported across the lakes) does suggest a distinct ecological niche adaptation, or in Baas Becking's words “the environment selects” for the specialist OTUs, as opposed to a tolerance over a broad range of ecological parameters for the generalist OTUs. Future studies focussing on the physiologically active microbial communities in each lake should shed light on this question and help to identify true “generalists.”

### Spatial and temporal diversity patterns

There is a distinct seasonal shift in the community composition of the lakes with no overlap of the summer and winter samples on the NMDS plot (Figure 10). Temperature is an obvious difference between summer and winter environmental conditions. However, if the shift in community composition was driven by temperature alone, the winter samples should cluster closer together than the summer samples, since the temperature gradient along the chain of lakes is far less pronounced in winter. Instead, they are—like the summer samples—spread out following the geographical position of the lakes as well as the gradients of physical and chemical parameters, indicating that in both summer and winter the community composition is governed by the gradients of environmental conditions along the chain of lakes. CCA (Figure 8) shows that the driving environmental parameters in these gradients differ among the lakes. The group I lakes are more influenced by gradients of phosphate, nitrate and conductivity, while the group IV lakes are mainly influenced by temperature, ammonia and oxygen. The lakes of groups II and III show only weak correlations with the measured environmental parameters.

Cyanobacteria and eukaryotic phytoplankton show a contrasting spatial distribution in the Osterseen Lake District, with eukaryotic sequences dominating the eutrophic lakes and cyanobacteria sequences the oligo-mesotrophic ones. The almost complete absence of cyanobacteria in the eutrophic lakes seems counter-intuitive at first, since there is a well-documented positive correlation between cyanobacteria, particularly harmful bloom-forming species, and high nutrient loads (Fogg, 1969; Chorus and Bartram, 1999; Paerl and Otten, 2013). However, temperature, rather than nutrients may be the decisive factor in these lakes. The eutrophic Lake Waschsee, Lake Schiffhüttensee and Lake Sengsee all have a year-round steady influx of cold (8–10°C) ground water. Due to this, they generally do not freeze over in winter, but their average temperature throughout summer barely rises above 15°C. Cyanobacteria generally thrive at higher temperatures, whereas eukaryotic phytoplankton have lower temperature optima (Butterwick et al., 2005; Elliott et al., 2006; de Senerpont Domis et al., 2007; Jöhnk et al., 2008), a finding that has led to predictions of an increase of cyanobacteria and harmful cyanobacterial blooms due to climate change (Elliott, 2012; El-Shehawy et al., 2012; Paerl and Paul, 2012). A further consequence of the ground water influx is the slightly lower pH in the first three lakes of ca. 7.0–7.5 as opposed to >8.0 in the other lakes, which could also favor the growth of eukaryotic phytoplankton rather than cyanobacteria (Caraco and Miller, 1998). Contrary to bloom-forming cyanobacterial genera associated with high nutrient conditions, picocyanobacteria, such as *Synechococcus*, can thrive in oligotrophic conditions (Sherr et al., 2005) and have recently been shown (in a marine study) to outcompete larger eukaryotic phytoplankton in oligotrophic conditions (Zubkov, 2014). The vast majority of cyanobacteria in the Osterseen Lake District have been classified as *Synechococcus*. However, not all *Synechoccoccus* strains are adapted to low nutrient conditions. Members of the marine *Synechococcus* clade II are predominantly found in meso-eutrophic coastal waters (Zwirglmaier et al., 2007, 2008). Whether there is a corresponding high-nutrient-adapted *Synechococcus* clade in freshwater systems is currently unclear, as niche-adaptation in freshwater picocyanobacteria is still poorly understood.

Several lakes of the Osterseen Lake District that stand out in their physical and chemical properties compared to the other lakes, also had distinctly different microbial community compositions. These are Lake Waschsee and Lake Schiffhüttensee on the one hand and Lake Eishaussee on the other hand.

The eutrophic, cold and rather shallow Lake Waschsee and Lake Schiffhüttensee, which are very similar to each other in their microbial community composition, are notable not only due to their lack of cyanobacteria. They are also completely devoid of Planctomycetes and have much lower numbers of Verrucomicrobia sequences than the other lakes. Instead, they are dominated by a range of Bacteroidetes, particularly of the genera *Flavobacterium, Flaviicola* and *Pseudarcicella*. *Pseudarcicella*, with its only described species to date *P. hirudinis* has been isolated from the skin of the medical leech *Hirudo medicinalis* (Kämpfer et al., 2012). While we are not aware (but cannot exclude the possibility) of the presence of leeches in these lakes, the presence of considerable numbers of *P. hirudinis* sequences in the water column here indicates that this organism (or rather the particular OTU found here) is not restricted to epibiotic growth on leeches. In fact, the current SILVA database (r123) contains a large number of sequences of uncultured bacteria with >99% identity with *Pseudarcicella*, that were detected in a wide range of habitats, including lakes, rivers, estuary, sediment, and waste water.

The microbial community in the mesotrophic Lake Eishaussee (western and eastern basin) stands out from the other lakes due to the dominance of a range of anaerobic photolithotrophic (*Chlorobium*, Pfennig and Overmann, 2001), sulfate-reducing (*Desulfatirhabdium*, Balk et al., 2008) and aromatic compound-degrading (WCHB1-69, Dojka et al., 1998) bacteria. They are likely to originate from the permanently anoxic bottom layer of the meromictic western basin of the lake, which has a strong smell of H_2_S throughout the year. The presence of the same bacteria in the dimictic eastern basin of the lake, which lacks an anoxic, sulfide-rich layer, indicates a certain amount of water exchange between the basins, although overall they only share 37.6% of their OTUs and have a Bray-Curtis similarity of 0.54. This similarity is high compared to Lake Fischkaltersee and Lake Herrensee, which share only 20.9% of their OTUs with a Bray-Curtis similarity of just 0.14 even though they are directly adjacent. They were heavily dominated by three OTUs of Gammaproteobacteria, classified as *Rheinheimera* (in lake Herrensee), *Aeromonas* and *Acinetobacter* both in lake Fischkaltersee), which only appear in these two lakes. There was surprisingly little crossover of these OTUs between the lakes with only 0.9% of *Rheinheimera* found in lake Fischkaltersee and 6.1% of *Aeromonas* and 1% of *Acinetobacter* found in lake Herrensee, indicating a strong adaptation to the prevalent environmental conditions in the respective lakes. Lake Herrensee has always been classed as oligotrophic in the last three decades, which is in line with the preferences described for *Rheinheimera texasensis* (Merchant et al., 2007), the closest relative to the OTU found here (99.2% sequence identity). Lake Fischkaltersee, on the other hand, has undergone significant changes in that time. A eutrophic lake in the 1970's and 80's, it had developed a haline, anoxic bottom layer due to wash-off of gritting salt from the nearby road. Phosphate precipitation with aluminum hydroxide in 1979 and later artificial aeration in 1980, as well as further environmental protection measures in the surrounding area led to a continuous improvement of the water quality and it has now reached a meso-oligotrophic status. The high abundance of *Aeromonas* and *Acinetobacter* still point to the eu-mesotrophic past of the lake, even though the phosphate concentrations of <5 μg/L at the time of sampling seem to indicate oligotrophic nutrient conditions. Both genera are frequently found in fresh and brackish waters, but also in waste water (Towner et al., 1991; Martin-Carnahan and Joseph, 2005). Many species within those genera are known pathogens, although due to the limited length of the sequence reads there is no conclusive evidence whether the OTUs found in this lake are pathogenic.

### Fine-scale biodiversity

Since the rise of next generation sequencing (NGS) within the last decade, microbial biodiversity studies have increased exponentially. However, most of these studies to date compare samples only at the level of bacterial phyla. As we have shown here, this approach may mask distinct patterns in microbial distribution. This is particularly important given the vast physiological differences of bacterial species that are classed within the same phylum or even the same genus. Striking examples to illustrate this are *Clostridium tetani* and *Lactobacillus acidophilus*, both members of the Firmicutes. The former is an obligate anaerobe toxin-producer that causes tetanus, while the latter is microaerophilic and used as a starter culture in yogurt production. Within the genus *Vibrio* (Gammaproteobacteria) *V. cholerae* is a lethal pathogen, while *V. fischeri* is a non-pathogenic, symbiotic bioluminescent species. Both are found in aquatic habitats. In our dataset, we found that OTUs classed within the same phylum can exhibit a wide range of positive or negative correlations with various environmental parameters (Figure 9) suggesting adaptation to different ecological niches and therefore putatively different physiological capabilities. Another example is the dominance of Gammaproteobacteria in the directly adjacent Lake Fischkaltersee and Lake Herrensee, which at the level of OTUs were revealed to be different genera of Gammaproteobacteria. They have apparently (based on the measured physical and chemical parameters in the respective lakes) very different environmental requirements and presumably (based on what is known about other closely related cultivated species within those genera) very different physiological capabilities.

One reason for presenting NGS biodiversity data only at the level of phyla used to be the read length of NGS sequence data. The first generation of 454 machines only achieved read lengths of ca 100 nt (Margulies et al., 2005), Illumina started off with 35 nt (Bentley et al., 2008), which prevented reliable classification beyond phylum level. However, current technologies provide read lengths of several hundred nucleotides, e.g., 2 × 300 nt paired ends for Illumina MiSeq and up to 1000 nt for 454, at which point ignoring diversity beyond phylum level seems like wasting valuable information. In the presented study we used sequence reads trimmed to 500 nt, covering the variable regions V5–V8 of the 16S rRNA gene. This is certainly long enough for a definite classification of the sequences according to phylum and in most cases also class, family and genus, but not necessarily species (Yarza et al., 2014). However, sequences can be reliably clustered into OTUs at the level of 97% sequence identity, i.e., two OTUs have at least 15 differences in their 500 nt sequence. What is not possible, but also not strictly necessary, is attributing a specific “species name” to these OTUs. These patterns would have been missed entirely at the phylum level. We therefore strongly advocate a resolution at OTU level for future biodiversity studies.

As demonstrated in this study, knowledge on niche adaptations and ecological preferences of microbiota in relation to physicochemical lake properties is essential in determining and understanding their local occurrences. Such knowledge is not only relevant for ecologists, but must also be seen in the context of the many human uses of lake ecosystems. In light of anthropogenic changes such as global warming or changes related to altered nutrient status, the current focus on macroscopic biodiversity responses (reviewed e.g., in Geist, 2014, 2015) needs to be expanded by also including the microbial level.

### Conflict of interest statement

The authors declare that the research was conducted in the absence of any commercial or financial relationships that could be construed as a potential conflict of interest.

## Supplementary material

Supplementary Table ST 1 **Full OTU table of all bacterial OTUs (excluding chloroplasts), abundance in percentage, corrected with PICRUSt including taxonomic classification and % identity with closest relative**.
